# Draft Genome Sequence of *Mycobacterium bovis* Strain D-10-02315 Isolated from Wild Boar

**DOI:** 10.1128/genomeA.01268-16

**Published:** 2016-11-10

**Authors:** Maxime Branger, Amandine Hauer, Lorraine Michelet, Claudine Karoui, Thierry Cochard, Krystel De Cruz, Sylvie Henault, María Laura Boschiroli, Franck Biet

**Affiliations:** a INRA, Université de Tours, UMR1282, Infectiologie et Santé Publique, Nouzilly, France; b Université Paris-Est, Laboratoire National de Référence Tuberculose, Unité Zoonoses Bactériennes, Laboratoire de Santé Animale, ANSES, Maisons-Alfort, France

## Abstract

*Mycobacterium bovis* is the etiologic agent of bovine tuberculosis, a chronic infectious disease, affecting livestock, wild animals, and sometimes humans. We report the draft genome sequence of a *Mycobacterium bovis* strain isolated from wild boar of spoligotype SB0120 (or BCG-like) also present in wildlife-livestock multi-host systems.

## GENOME ANNOUNCEMENT

*Mycobacterium bovis* is the causal agent of bovine tuberculosis (BT), a chronic infectious disease, affecting domestic animal species like cattle, wild animals, and sometimes human beings (1–4). France is an officially BT-free EU member, although every year several outbreaks occur in a few regions of the country (5).

The description of the population of *M. bovis* circulating in France for the last 35 years highlighted that strains of spoligotype SB0120 (or BCG-like), comprising up to 53 different MIRU-VNTR genotypes (4, 6), are the most common. These genotypes of strains are widespread all over the country but, in the last 10 years, a few specific MLVA-profiles seem to spread in particular regions (7). In Dordogne (southwest of France), the main *M. bovis* strain isolated from infected animals is SB0120−MLVA 5 3 5 3 9 4 5 6. This strain is present in wildlife-livestock multi-host systems, including wild boar, badgers, roe deer, and cattle (7, 8).

We performed whole-genome sequencing of *M. bovis* in order to improve the knowledge of the genetic determinants involved in modulation of virulence, spread in multi-host systems and persistence over time. The strain SB0120−MLVA 5 3 5 3 9 4 5 6 was isolated in Dordogne in 2010 from mandibular lymph nodes of a wild boar as described before (7).

The isolate was grown in 10 mL of Middlebrook 7H9 liquid media (Becton-Dickinson, France) supplemented with 10% OADC (oleic albumin dextrose catalase) for 4 weeks. Bacteria were thermalized for 1 h at 80°C and chromosomal DNA was extracted by phenol-chloroform method.

DNA sequencing was performed at Genoscreen (Lille, France) on an Illumina HiSeq 2500 (Illumina, San Diego, CA, USA), to get 2 × 100 paired-ends sequences for an average coverage of 80×. Sequencing produced 1,929,897 paired-ends reads of 100 bp and 1,844,142 reads after filtering with sickle version 1.33 (9). We performed an alignment between filtered reads and the *M. bovis* AF2122/97 strain as reference using bowtie2 version 2.2.6 (10). The alignment gave us 3,670,788 reads mapped on the reference corresponding to 97.28% coverage. We then performed *de novo* assembly using Spades version 3.9.0 (2) on trimmed sequences with a k-mer size of 55. We obtained 135 contigs (the largest one being 205,553 bp) for a total length of 4,295,676 bp (using only contigs larger than 500 bp), a G+C content of 65.55%, and an *N*_50_ of 72,210 bp (QUAST version 4.2 [11]). Annotation was performed by the National Center for Biotechnology Information (NCBI) Prokaryotic Genome Annotation Pipeline (12) which predicted 3,842 coding DNA sequences (CDS) for 4,073 total genes, 45 tRNAs, and 180 pseudo-genes.

### Accession number(s).

This whole-genome shotgun project has been deposited at DDBJ/ENA/GenBank under the accession number MINA00000000. The version described in this paper is version MINA01000000.
